# Heat-mediated reduction of apoptosis in UVB-damaged keratinocytes in vitro and in human skin ex vivo

**DOI:** 10.1186/s12895-016-0043-4

**Published:** 2016-05-26

**Authors:** Leslie Calapre, Elin S. Gray, Sandrine Kurdykowski, Anthony David, Prue Hart, Pascal Descargues, Mel Ziman

**Affiliations:** School of Medical Sciences, Edith Cowan University, 270 Joondalup Drive, Joondalup, Perth, WA 6027 Australia; GENOSKIN Centre Pierre Potier, Oncopole, Toulouse, France; Department of Pathology and Laboratory Medicine, University of Western Australia, Crawley, WA Australia; Telethon Kids Institute, University of Western Australia, 100 Roberts Road, Subiaco, Perth, 6008 Australia

**Keywords:** Heat stress, UVB, Keratinocytes, Apoptosis, p53, DNA damage

## Abstract

**Background:**

UV radiation induces significant DNA damage in keratinocytes and is a known risk factor for skin carcinogenesis. However, it has been reported previously that repeated and simultaneous exposure to UV and heat stress increases the rate of cutaneous tumour formation in mice. Since constant exposure to high temperatures and UV are often experienced in the environment, the effects of exposure to UV and heat needs to be clearly addressed in human epidermal cells.

**Methods:**

In this study, we determined the effects of repeated UVB exposure 1 kJ/m^2^ followed by heat (39 °C) to human keratinocytes. Normal human ex vivo skin models and primary keratinocytes (NHEK) were exposed once a day to UVB and/or heat stress for four consecutive days. Cells were then assessed for changes in proliferation, apoptosis and gene expression at 2 days post-exposure, to determine the cumulative and persistent effects of UV and/or heat in skin keratinocytes.

**Results:**

Using ex vivo skin models and primary keratinocytes in vitro, we showed that UVB *plus* heat treated keratinocytes exhibit persistent DNA damage, as observed with UVB alone. However, we found that apoptosis was significantly reduced in UVB *plus* heat treated samples. Immunohistochemical and whole genome transcription analysis showed that multiple UVB *plus* heat exposures induced inactivation of the p53-mediated stress response. Furthermore, we demonstrated that repeated exposure to UV *plus* heat induced SIRT1 expression and a decrease in acetylated p53 in keratinocytes, which is consistent with the significant downregulation of p53-regulated pro-apoptotic and DNA damage repair genes in these cells.

**Conclusion:**

Our results suggest that UVB-induced p53-mediated cell cycle arrest and apoptosis are reduced in the presence of heat stress, leading to increased survival of DNA damaged cells. Thus, exposure to UVB and heat stress may act synergistically to allow survival of damaged cells, which could have implications for initiation skin carcinogenesis.

**Electronic supplementary material:**

The online version of this article (doi:10.1186/s12895-016-0043-4) contains supplementary material, which is available to authorized users.

## Background

The anatomical location of epidermal keratinocytes makes them vulnerable to the effects of UV radiation and temperature fluctuations [1]. UV radiation, particularly UVB, can induce DNA damage, in the form of cyclobutane pyrimidine dimers (CPD), and is a known risk factor for skin carcinogenesis [2, 3]. Moreover, it is well-known that UV irradiation of normal keratinocytes activates the p53 tumour suppressor protein, which is crucial for regulating cell cycle arrest, apoptosis and nuclear excision repair of UV-induced DNA damage [4–8].

Heat stress also causes DNA damage and has been observed to deaminate cytosine and hydrolise glycosyl bonds, leading to genome instability [9–11]. In addition, exposure to heat stress can induce formation of reactive oxygen species, which can, cause G to T transversion mutations [11–14]. Heat stress can also trigger extensive denaturation, degradation and aggregation of critical intracellular proteins [15, 16], leading to defective DNA replication, transcription and repair, thus affecting cell survival and apoptosis [17–19]. The deleterious effect of heat on cellular processes is normally countered by activation of a conserved heat shock response [20–22], which stabilises cells by interacting with pro-survival signalling pathways such as PI3K/Akt [20, 23, 24].

Continuous exposure to heat stress independently or concurrently with UV radiation is commonly experienced in several geographical locations. Furthermore, a previous report has shown increased risk of non-melanoma skin cancers in geographical areas with high environmental temperature [25]. However, little work has been done to show the consequences of repeated exposure of keratinocytes to both high temperatures and UV [26]. Previous studies have shown that pre-treatment with heat shock at 38–42 °C, prior to UVB irradiation, increases cell survival and decreases UVB-induced DNA damage of normal murine and human keratinocytes [27–30]. However, it is important to note that these previously described experiments involved a singular exposure to heat then UV. Other studies have shown that repeated and simultaneous exposure to UVB and heat stress increased the rate of cutaneous tumour formation in mice [31, 32]. Thus, the effects of multiple simultaneous exposures to heat and UVB need to be clearly addressed in human keratinocytes, and molecular changes in response to UVB *plus* heat remain to be characterised.

In this study, we investigated the effects of heat stress alone, or immediately after UVB radiation, on primary keratinocyte cultures in vitro and in an ex vivo human skin model. Given that exposure to UVB radiation and/or heat stress is often repeatedly experienced in nature, we particularly aimed to determine the effects of multiple exposures to these environmental stressors. Thus, to determine whether heat alleviates or exacerbates the effects of UVB irradiation, we looked at the level of DNA damage, apoptosis and cell proliferation in keratinocytes two days after repeated UVB and/or heat treatment. To detail the molecular events underpinning the observed cellular changes, a whole genome gene expression array was performed in heat and/or UVB treated samples, and pathways activated by UVB *plus* heat were identified. Moreover, we investigated the expression of key proteins involved in the affected molecular pathways activated in DNA-damaged cells.

## Methods

### Cell lines

Primary adult human epidermal keratinocytes (NHEK-c, Promocell) were cultured in vitro using Keratinocyte Growth Medium 2 (Promocell) supplemented with CaCl_2_ (0.06 mM) and penicillin/streptomycin (Sigma-Aldrich, AUSTRALIA).

### Skin model

NativeSkin® (Genoskin, France) models are ex vivo punch biopsies of normal human skin embedded in a matrix and fixed in a cell culture insert. Twelve skin models were generated from non-sun exposed skin of a donor. The skin biopsies were reported as clear of any lesions. Informed consent from donors and ethics approval was obtained for commercialisation and experimental use of the skin biopsies.

### UVB radiation and heat exposure

A UV cabinet fitted with a TL20W/01 RS SLV Narrowband UVB lamp (Philips, GERMANY), with a spectral output between 305–315 nm, was used to administer UVB irradiation at a dose of 1 kJ/m^2^. Cells were covered with a thin layer of pre-warmed PBS (37 °C) and ex vivo skins were maintained in their nourishing matrix during the irradiation process. PBS was removed and replaced with culture immediately following UVB exposure. Heat stress involved culture in a normal CO_2_ incubator, with temperature maintained at 39 °C for three hours. The temperature used in the experiments was based on previous measurements of skin surface temperature of miners, who are prone to intense heat stress, in the Pilbara region of Western Australia (unpublished data). For UVB *plus* heat exposures, cells and skin models were exposed to 1 kJ/m^2^ of UVB followed by 3 h of heat stress (39 °C) once per day, for four consecutive days. Cell proliferation, apoptosis and whole genome expression profiles were analysed two days after the last exposure.

To analyse proliferation and apoptosis, primary keratinocytes at passage 4–6 were seeded in a 6-well plate at 200,000 cells/well, and in LabTek Chambered Microscopic slides (Thermofisher, AUSTRALIA) at 100,000 cells/well for immunocytochemistry analysis. Cells were at 50 % confluence at the point of first UVB and/or heat exposures. Each experiment was performed in triplicate and each set of experiments included untreated cells which underwent similar handling.

For the skin model experiments, NativeSkin® was placed in a 6-well plate provided with media and then incubated overnight prior to initial UVB and/or heat exposures. Experiments were performed in duplicate. Untreated skins underwent similar processing and handling to the treated skins but were not exposed to any UVB radiation and were kept at 37 °C throughout the experiments. These samples were considered experimental controls.

### Cell count and apoptosis assay – NHEK

Treated primary NHEK cells were trypsinised two days post UVB and/or heat exposure, centrifuged at 300 g for 5 min, resuspended in 500 μL media, and counted in a Vi-Cell™ Viability Analyser (Beckman-Coulter). The level of apoptosis for exposed primary cells was determined using Annexin V-FITC Apoptosis Detection Kit I (BD Pharmingen, USA) and cells were stained as per the manufacturer’s instructions. Samples were analysed using the Gallios™ flow cytometer (Beckman-Coulter). For each sample, 10,000 events were acquired. Annexin V^+^PI^−^cells represented early apoptotic populations and Annexin V^+^PI^+^ cells represented either late apoptotic or secondary necrotic populations.

### Immunohistochemistry – skin proliferation and apoptosis

For immunohistochemistry, the formalin-fixed paraffin-embedded (FFPE) skin tissues were de-waxed at 58 °C for 20 min, and deparaffinised with xylene and hydrated with a graded series of ethanol. Sections were microwaved for 15 min in sodium citrate buffer for antigen retrieval, permeabilised with 0.025 % Triton-X, blocked with 1 % BSA in TBS and incubated with antibodies either thymine dimer/cyclobutane pyrimidine dimer (CPD) (mouse monoclonal, 1:500 dilution; Kamiya Biomedical, USA), active-caspase-3 (rabbit monoclonal, 1:50 dilution; Abcam, USA), p53 antibody (rabbit monoclonal, 1:50 dilution; Abcam, USA), acetylated p53-382 (rabbit polyclonal, 1:250 dilution, Abcam, USA), SIRT1 (mouse monoclonal, 1:250 dilution, Abcam, USA) or SIRT1-p (rabbit monoclonal, 1:250). Anti-pan-cytokeratin (mouse monoclonal 1:50; Abcam, USA) was used to label keratinocytes. Anti-mouse Alexa Fluor 550 and anti-rabbit Alexa Fluor 488 secondary antibodies were used for detection. The stained tissues were mounted using Prolong Gold Mounting media with DAPI.

To determine percentages of keratinocytes positive for certain markers, three sections, which are considered as experimental triplicates, were analysed per biological duplicate (*n*=2 × 3-sections) and five random fields-of-view were quantified to generate a value of positive cells per section. The number of cells in each of the 6 sections were analysed for standard errors.

### Ex vivo gene expression analysis

RNA was isolated from skin tissues using an AllPrep RNA/DNA Mini Kit (SABioscience, AUSTRALIA). RNA extracted from samples was sent to the Australian Genome Research Facility (Melbourne, Australia) where it was reversed transcribed to cDNA and hybridised to the Human HT-12 Expression v4 BeadChip (Illumina, USA) for whole genome expression profiling. This microarray targets 47,231 probes derived from genes in the NCBI RefSeq database. The relative expression of each gene was calculated as fold-change and *p*-value relative to untreated tissue using R version 3.1.1. Ingenuity Pathway Analysis (IPA) (Qiagen, USA) annotated the effects of altered gene expression on cell function and upstream signalling pathways. Significant transcription regulators and cellular functions were identified as enriched and significant using fold changes and p-values.

### Statistical analyses

Two-way ANOVA was used to analyse differences across treatment groups, while parametric unpaired t-tests were used to detect differences between specific treatment groups in all experimental categories, i.e. proliferation, apoptosis and gene expression, with *p*-values <0.05 considered significant.

## Results

### UVB *plus* heat exposed keratinocytes exhibit persistent DNA damage but significantly less apoptosis in vitro

**Fig. 1:**
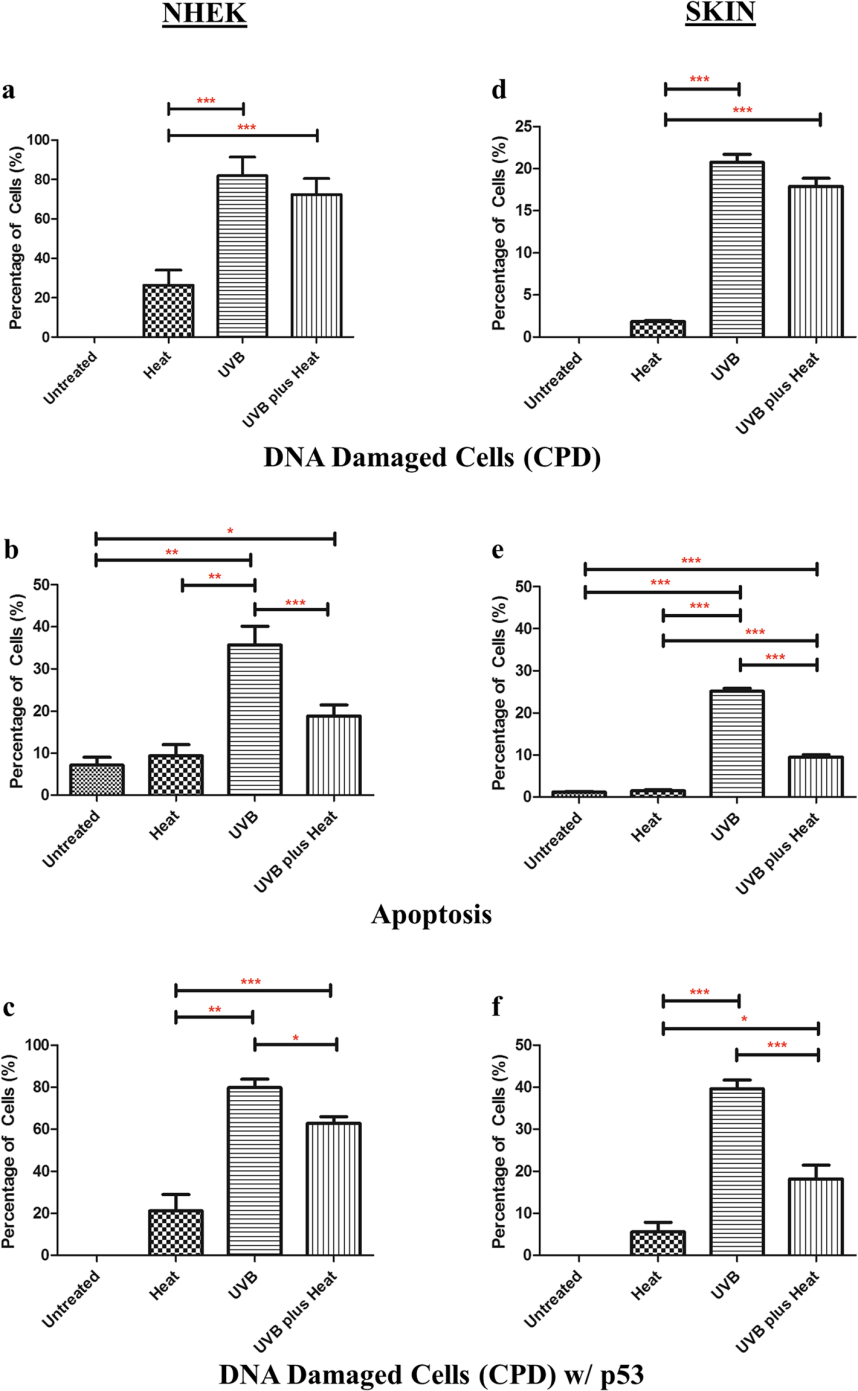
Effects of UVB and/or heat stress on DNA damage and apoptosis in keratinocytes in vitro and in an ex vivo skin model. Bar graphs of mean ± SD percent keratinocytes that (a and d) harboured DNA damage (CPD), (b and e) were apoptotic, or (c and f) harboured DNA damage and expressed p53 in (**a**–**c**) NHEK or (**d**–**f**) skin per field of view. Statistically significant differences are indicated with *, ** or *** for *p*-values <0.05, <0.01 or ≤0.001 respectively

We first examined the presence of DNA damage in UVB and/or heat exposed keratinocytes by labelling the DNA with anti-thymine dimer antibody (CPD). The number of DNA damaged cells (CPD-positive keratinocytes) was calculated as a percentage of the total number of keratinocytes per field of view (representative pictures of the IHC staining for each sample are shown in Figure S1). None of the untreated cells had detectable CPD, while 26 ± 7 % (mean ± SD) of heat treated keratinocytes had DNA damage (Fig. 1a). By contrast, 83 ± 9 % and 72 ± 5 % of keratinocytes showed DNA damage in UVB and UVB *plus* heat treated samples respectively, which was significantly greater than the number of CPD-positive cells in heat exposed keratinocytes (*p* ≤ 0.001for both).

We next quantified the number of cells surviving after multiple exposures to UVB and/or heat, and also determined what proportion of these were apoptotic and/or necrotic cells (Fig. 1b and Table S1). Heat treated keratinocytes had a slightly higher cell count (5.4 × 10^6^) and higher percentage of apoptotic cells (9 ± 4 %) than untreated cells (4 × 10^6^ and 7 ± 3 % respectively). By contrast, UVB treated cells had the lowest cell count (1.15 × 10^6^), with significantly higher number of necrotic (50 ± 4 %) and apoptotic (35 ± 8 %) cells relative to untreated (*p*=0.004) or heat treated (*p*=0.02) samples. Remarkably, UVB *plu*s heat treated keratinocytes exhibited a higher cell count (2.25 × 10^6^) and significantly reduced numbers of necrotic (23 ± 5 %, *p*=0.04) and apoptotic cells (19 ± 4 %, *p* ≤ 0.001) relative to those treated with UVB alone. Thus, heat appears to diminish the level of cell death induced by UV irradiation. Overall, these results show that multiple consecutive exposures to UVB followed immediately by heat stress reduce apoptosis and necrosis, and increases survival of keratinocytes.

### Primary keratinocytes treated with UVB *plus* heat showed diminished p53 response in vitro

We then assessed the presence of p53 in keratinocytes showing persistent DNA damage, to determine the efficiency of the cellular stress response in these cells (Fig. 1c). The number of p53/CPD-positive keratinocytes was calculated as a percentage of the total number of CPD-positive keratinocytes per field of view. In the heat treated samples, approximately 21 ± 8 % of CPD positive cells were also p53 positive. By contrast, 83 ± 6 % of CPD-positive keratinocytes in UVB irradiated samples expressed p53, confirming that repeated UVB treatment activates this cellular stress response [6]. Interestingly, despite having similar numbers of CPD positive cells as UVB exposed cells, only 63 ± 4 % of UVB *plus* heat treated cells were p53-positive, which was significantly lower than that of UVB irradiated samples (*p*=0.01).

### UVB *plus* heat exposure induced DNA damage but lower numbers of apoptotic keratinocytes in an ex vivo skin model

Given the observed changes in apoptosis and cell survival after UVB *plus* heat stress in primary NHEK cells in vitro, we next examined if similar effects were induced in these cells in an ex vivo human skin model. As in the in vitro experiments, we first looked at the level of DNA damage and apoptosis in exposed skin samples by labelling with antibodies to CPD (Fig. 1d). Untreated skins were found to be negative for CPD, while 2 ± 1 % of heat treated epidermal keratinocytes had DNA damage. After multiple exposures, UVB and UVB *plus* heat treated epidermal keratinocytes had significantly higher numbers of cells with DNA damage, at 23 ± 1 % and 18 ± 2 % respectively (*p*=0.001 for both).

To determine the level of apoptosis after several exposures to UVB and/or heat, we labelled epidermal keratinocytes with cleaved (active) caspase-3 protein, a marker of apoptosis (Fig. 1e). Approximately 1 ± 1 % of keratinocytes were apoptotic in both the untreated and heat treated skins. As expected, UVB irradiated skins showed a significant increase (*p* ≤ 0.001) in the number of apoptotic keratinocytes (24 ± 1 %) relative to untreated and heat treated samples. UVB *plus* heat treated skins had significantly less apoptotic keratinocytes (9 ± 1 %) than UVB irradiated samples (*p* ≤ 0.001), suggesting possible impairment of apoptosis response mechanisms in UVB *plus* heat treated skin samples, as observed in NHEK cells in vitro.

### UVB *plus* heat exposure diminished cellular stress responses in DNA damaged epidermal keratinocytes

We next assessed the presence of p53 in epidermal cells with DNA damage to determine the efficiency of the cellular stress response in these cells (Fig. 1f). In the heat treated skins, among the 2 ± 1 % CPD-positive cells, approximately 5 ± 6 % were p53 positive. By contrast, 39 ± 2 % of CPD-positive keratinocytes in UVB irradiated samples expressed p53, suggesting that significantly higher proportions of damaged cells have an active cellular stress response, even at 2 days after multiple exposures. Interestingly, and as observed in vitro, the percentage of p53-positive keratinocytes with DNA damage (18 ± 3 %) was significantly lower in UV *plus* heat treated skins than in UV irradiated samples (*p*=0.006). These results suggest that the efficiency of the p53-mediated apoptosis response to DNA damage is diminished when UVB treatment is followed by heat exposure.

### UVB and UVB *plus* heat differentially affect expression of the genes involved in proliferation/survival and apoptosis in epidermal keratinocytes

To further understand the observed effects of multiple exposures to UVB *plus* heat and to characterise molecular events that govern such changes, we analysed the whole genome expression profiles of the exposed skin samples. Gene expression fold changes and *p*-values were calculated for each sample relative to untreated controls and considered significant when fold changes were ≥2 or <0.5 relative to untreated control samples and *p*-values were <0.05. Heat treated skin samples had only 7 significantly differentially expressed genes relative to controls. By contrast, UVB irradiated skins had 629 differentially expressed genes, while UVB *plus* heat treated samples had 4966.

In order to determine the biological significance of the observed changes in gene expression, we reviewed the data through the use of Ingenuity Pathway Analysis (IPA) software (Table S2). Due to the low number of affected genes, the functional annotations observed in heat treated samples were not significant. UVB irradiated skin samples showed significant upregulation of apoptosis related genes (*p* ≤ 0.001) and a significant downregulation of genes involved in cell viability (*p* ≤ 0.001) relative to untreated samples. Notably, UVB *plus* heat treated samples showed a significant downregulation in the expression of genes associated with apoptosis (*p* ≤ 0.001), as well as an increase in expression of cell viability (*p*=0.001) and mismatch repair of DNA (*p*=0.004) related genes.

**Fig. 2:**
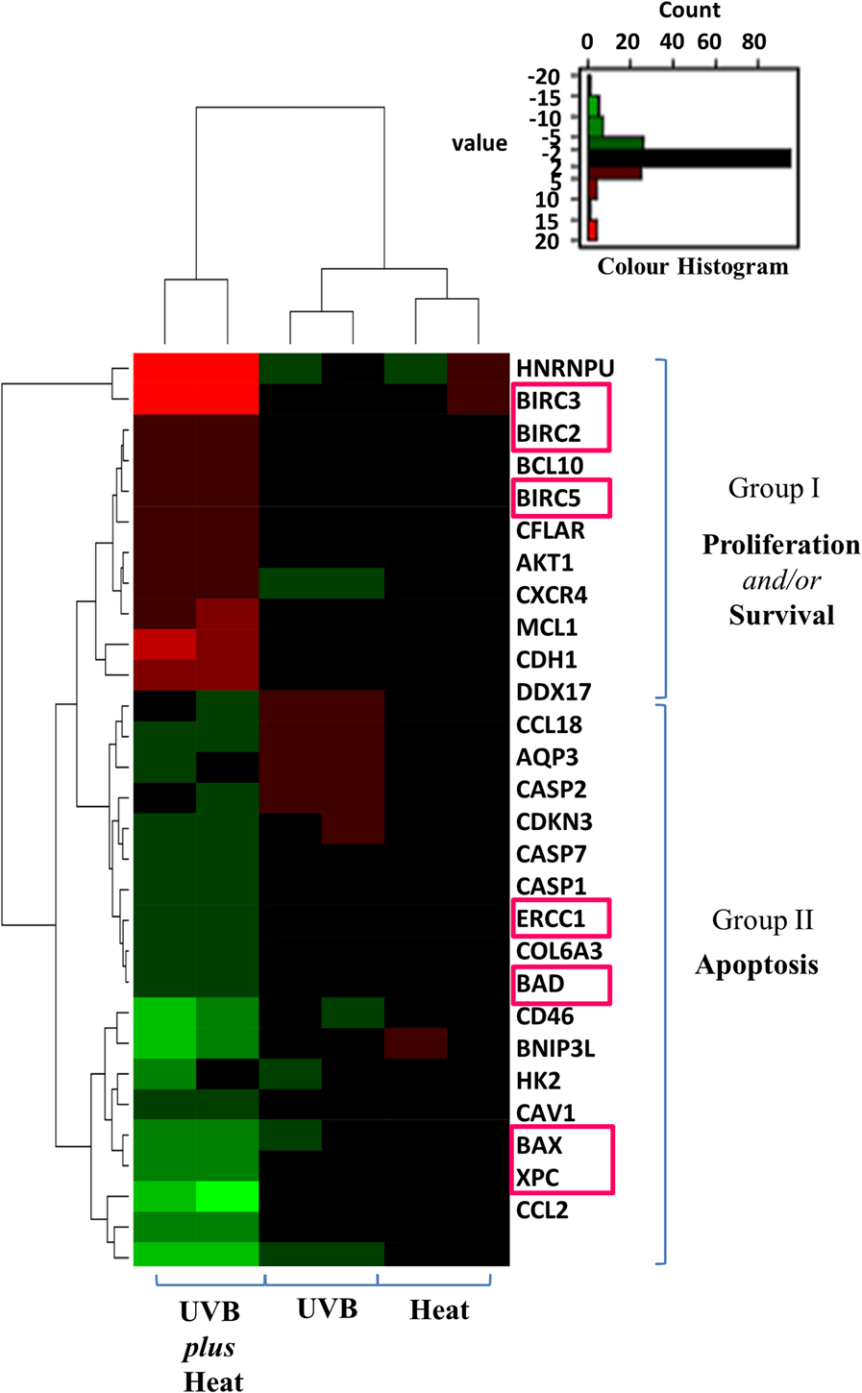
Exposure to UVB radiation and heat stress induced expression of genes with roles in cell survival. Hierarchal clustering analysis of 28 differentially expressed genes associated with biological changes observed in UVB and/or heat exposed ex vivo keratinocytes. Upregulation of cell-survival associated genes (red) and downregulation of apoptosis genes (green) are prominently clustered in UVB plus heat treated samples. Genes annotated by a pink box are known downstream targets of the p53 protein

We next focused on 28 genes found to be the most differentially expressed and associated with the functional pathways highlighted above. Hierarchical clustering of these genes revealed a clear segregation between UVB and UVB *plus* heat treated samples, and two distinct groups of genes became apparent (Fig. 2). Group 1 consisted of cluster of genes associated with the regulation of cell apoptosis, such as BAX and BAD, as well as DNA repair, such as XPC and ERCC1. These genes were downregulated in UVB *plus* heat but were either less affected or upregulated in UVB alone. By contrast, Group 2 was formed by genes associated with proliferation and survival pathways, particularly BIRC2, BIRC3 and survivin (BIRC5). These genes were upregulated in UVB *plus* heat but not affected or downregulated in UVB treated samples.

### Upstream regulatory factors important for cell survival are activated by UVB *plus* heat

Using IPA upstream analysis, we then examined the activity of key transcription factors and signalling pathways that govern cell apoptosis, survival and proliferation to determine if these were significantly affected. In particular, we looked for factors that would affect the observed gene expression changes in the activity of TP53, given its role in cell cycle regulation, apoptosis and DNA repair as well as the fact that damaged keratinocytes in UVB *plus* heat treated skins showed persistent significantly lower levels of p53 protein.

As expected, UVB exposed samples showed significant activation of cell cycle arrest and pro-apoptotic transcription regulators, particularly TP53 and CDKN1A (Table 1). By comparison, in UVB *plus* heat treated samples, TP53 activity was significantly reduced (*p* ≤ 0.001) and no upregulation of CDKN1A activity was apparent. In addition, classical upstream regulators of survival such as NFkB, ERK, PI3K and Akt were significantly inhibited in UVB exposed skin samples, but were not similarly affected in UVB *plus* heat. Likewise, HSF-1 was found highly activated only in UVB *plus* heat treated samples. We did not, however, observe HSF-1 activity after heat treatment alone; previous literature suggests that this gene is only upregulated at time points early after heat treatment [33]. Thus, consecutive exposure to UVB *plus* heat may have an enhanced effect on the activity of HSF-1 such that its activation was still apparent when measured 2 days after multiple consecutive treatments as performed in these experiments. Overall, our functional and upstream analysis confirms that cell cycle arrest and pro-apoptotic signalling were reduced after consecutive UVB and heat exposure, while proliferation and survival signalling were upregulated.

**Table 1:** Upstream regulators significantly activated in UVB *plus* heat

Upstream regulators	z score *(p value)*		Function	Reference
	UVB	UVB *plus* Heat		
*Pro-apoptotic signalling*				
TP53	3.25 *(4×10* ^*-6*^ *)*	-2.04 (*3.8×10* ^*-14*^)	Cell arrest and/or apoptosis	[8, 52]
CDKN1A	2.60 *(5×10* ^*-9*^ *)*		Inhibit cell proliferation and cell cycle progression	[53]
*Proliferation and Survival*				
NFkB (complex)	-2.87 *(2×10* ^*-4*^ *)*	-1.34 *(1×10* ^*-2*^ *)*	Cell survival	[54]
ERK	-2.42 (*7×10* ^*-3*^)	-1.77 (*1×10* ^*-3*^)	Cell survival and proliferation	[55, 56]
PI3K (complex)	-2.19 (*3×10* ^*-3*^)	-0.69 (*1×10* ^*-2*^)	Cell survival and proliferation	[57, 58]
Akt	-1.99 (*7×10* ^*-2*^)		Cell survival and proliferation	[59, 60]
*Others*				
HSF-1		1.98 (*2×10* ^*-8*^)	Heat shock response regulator	[37, 61]
SIRT1		2.16 (*8×10* ^*-4*^)	Cell proliferation and protection	[40]
Hdac (Histone deacetylases)		3.36 (2*×10* ^*-2*^)	p53 deacetylation and inhibition of nuclear translocation	[39, 62, 63]

### UVB *plus* heat induced upregulation of SIRT1 protein and a significant decrease in acetylated p53 in NHEK cells in vitro and in the skin models

SIRT1 is known to deacetylate p53 at lysine residue K382 of its c-terminal domain, which can diminish the ability of p53 to act as transcriptional modulator of its downstream gene targets [34–37]. We also observed persistent significant activation of histone deacetylases, particularly SIRT1, in our IPA analysis results (Table 1). Thus, we hypothesised that heat-induced interference in p53 signalling may be due to SIRT1-mediated deacetylation of p53.

Of note, phosphorylation of SIRT1 is important for the activation, stability and deacetylase function of the protein. Therefore, first we quantified the number of CPD containing keratinocytes that were positive for total (SIRT1) and phosphorylated SIRT1 (SIRT1-p) in vitro and in the skin model. We show that in fact all SIRT1 positive cells had the phosphorylated form of the protein in heat and UVB *plus* heat treated samples (Figure S2).

**Fig. 3:**
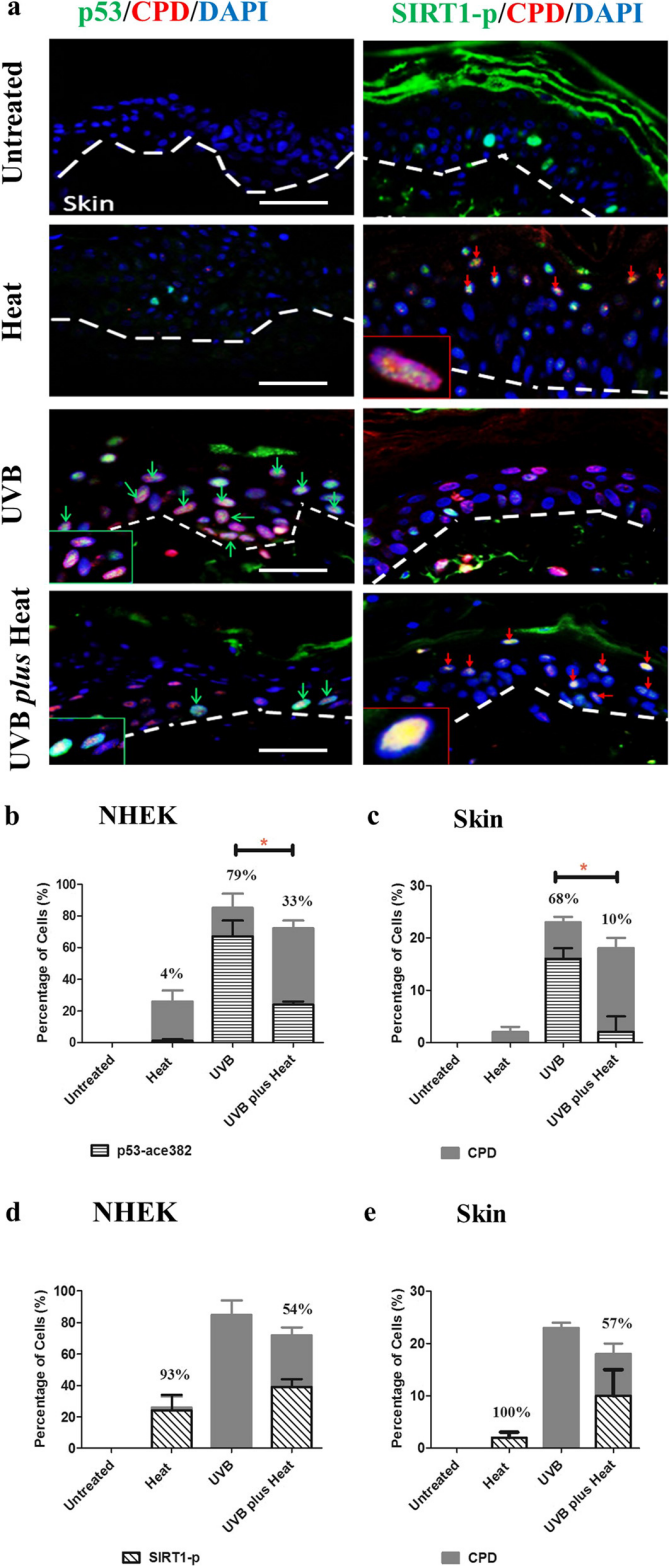
Exposure to UVB plus heat induced a significant decrease in acetylated p53 and an increase in SIRT1 protein levels in NHEK and in skin models. **a** Immunohistochemical staining of CPD (red), p53-a382 or SIRT1-p (green) and DAPI (blue) in untreated or UVB and/or heat treated ex vivo skin keratinocytes. Inset images are an enlarged view of SIRT1/DNA damaged (CPD positive) keratinocytes, which are also indicated by red arrows. Broken lines denote the epidermal/dermal border. Scale bar (white line) =100 μm. **b**–**e** Bar graphs of mean ± SD percent keratinocytes with CPD and positive for (**b** and **c**) p53-a382 or (**d** and **e**) SIRT1-p per field of view in (**b** and **d**) primary NHEK or (**c** and **e**) in skin models. Statistically significant differences are indicated with * for *p*-values <0.05

We, therefore, next assessed the number of CPD positive cells with p53 acetylated at lysine 382 (p53-a382). The number of p53-a382/CPD or SIRT1-p/CPD positive keratinocytes was calculated as a percentage of the total number of CPD-positive keratinocytes per field of view (Fig. 3). There was no CPD or acetylated p53 protein staining observed in the untreated primary NHEK cells in vitro (Figure S3). In the heat treated samples, only a small percentage (4 ± 1 %) of CPD-positive keratinocytes were positive for p53 acetylation in vitro (Fig. 3b), and none of the damaged cells were positive for acetylated p53 in skin keratinocytes (Fig. 3c). By contrast, 33 ± 2 % of damaged keratinocytes in UVB *plus* heat treated NHEK in vitro showed significantly lower p53 acetylation than in NHEK irradiated with UVB alone (79 ± 10 %, *p*=0.01). Similarly, in the skin models, DNA-damaged keratinocytes of the UVB *plus* heat treated samples showed significantly lower levels of acetylated p53 (10 ± 3 %) relative to UVB irradiated samples (68 ± 2 %, *p*=0.02).

Interestingly, in the heat treated samples, 93 ± 10 % of CPD-positive cells were positive for SIRT1-p in vitro (Fig. 3d), and all damaged cells showed SIRT1-p in the skin models (Fig. 3e). By contrast, 54 ± 5 % and 57 ± 5 % of damaged cells in UVB *plus* heat treated cells showed SIRT1-p protein, in vitro and in skin samples respectively. There was no SIRT1-p staining noted in UVB exposed cells. Noticeably, there was an overall negative relationship between the number of SIRT1-p/CPD positive cells and the number of acetylated p53/CPD positive cells in UVB *plus* heat treated samples. These results suggest that exposure to heat stress, in addition to UV, significantly inactivates p53 via SIRT1-mediated deacetylation of this protein.

## Discussion

In this study, we showed that the response to UVB-mediated cellular damage is diminished in the presence of heat and, for the first time, provide a molecular mechanism that explains these effects. NHEK cells in vitro and ex vivo epidermal keratinocytes that were repeatedly irradiated with UVB showed higher level of cellular damage and significant activation of cellular stress responses and pro-apoptotic signalling, evident by p53 activation and the high level of caspase-3, a protease involved in apoptosis of damaged cells [38–41], even at two days post-exposure. By contrast, keratinocytes with multiple heat and UVB exposures exhibited significant inactivation of the p53-mediated stress response and showed reduced numbers of apoptotic and necrotic cells at similar time point. Moreover, we show that SIRT1-mediated deacetylation of p53 is a possible mediator of these effects.

Previous studies have shown that singular pre-treatment with heat stress (38-41 ºC), prior to UVB (290–320 nm) exposure, increases viability and decreases thymine dimer formation in murine and human keratinocytes, suggesting heat-mediated protection of UVB-damaged keratinocytes [27–30]. By contrast, we found that with repeated exposure to UVB and high temperature, heat did not reduce DNA damage (presence of CPDs) but rather promoted the survival of keratinocytes containing these UVB-induced DNA lesions. Moreover, our results show that persistent heat-mediated survival and reduction of cell arrest, apoptosis and necrosis of damaged keratinocytes in UVB *plus* heat treated samples appears to be mediated by inactivation of p53 signalling.

The p53 protein is an important transcription factor involved in maintaining genome integrity upon exposure to UV, either by enforcing a G1 cell cycle arrest, inducing apoptosis or enhancing nuclear excision repair of damaged cells [6, 42]. While gene expression of TP53 was not significantly affected or the mRNA level reduced after multiple UVB and heat exposures, the expression levels of the majority of its downstream gene targets, particularly BAX and survivin, were consistent with inactivation of the p53 protein. As transcription of BAX and survivin is regulated via binding of p53 to their promoters [43, 44], this set of results suggests possible impairment of the DNA-binding capability of p53.

We also noted a significant activation of SIRT1 in UVB *plus* heat treated samples. The number of damaged cells with phosphorylated SIRT1 protein corresponded to the number of damaged cells that were negative for p53 acetylation at K382 in UVB *plus* heat treated samples. Thus, inactivation of p53 signalling, observed after multiple exposures to UVB and heat, may be attributed to the SIRT1-mediated post-translational modification of the p53 protein. SIRT1 is a histone deacetylase that can deacetylate p53 and significantly reduce its DNA binding capability, which can lead to deregulation of p53 dependent genes [8, 36, 45, 46]. It is important to note that SIRT1 activity is downregulated by UV [47, 48], but is consistently increased after heat shock [34–36]. Thus, heat stress may activate SIRT1 in damaged cells, which leads to deacetylation and inactivation of p53, and diminishing the capacity of the p53 protein to bind and regulate transcription of its downstream target genes.

The discrepancy between our findings and previous studies is likely a result of the differences in the exposure models. It is important to note that previous studies adhered to a single heat-then-UVB exposure experimental protocol, with a four-hour interval between heat and UVB exposures [28, 49, 50]. Pre-treatment to heat stress prior to UVB irradiation was shown to activate the heat shock response and increase expression of HSP70 protein in keratinocytes [17, 51], and thus these cells were thought to have been provided with a pre-established protective mechanism against UVB-induced DNA damage. By contrast, our study was based on repeated UVB and heat exposures, with heat exposure following immediately after UVB. In the environment, UVB and heat stress often occur concurrently and can, therefore, synchronously affect epidermal cells. Thus, the exposure model used here may have affected the ability of keratinocytes to mount an appropriate response to UVB-mediated DNA damage, presumably via impairment of p53-mediated cell apoptosis. In addition, the use of narrowband UVB instead of broadband UVB lamps may have contributed to the different factors activated in keratinocytes as a result of UVB *plus* heat exposure. This study was originally conceptualised to determine if extreme heat, in addition to UV, can have major effects on epidermal cell biology. Narrowband UVB was used in this study as it has previously been shown to induce higher frequency of skin cancer in mice [52], and thus was more fitting in creating an exposure model where the consequent UV- and/or temperature-induced damage are high.

Nevertheless, further studies are required to establish direct association between p53 and SIRT1 activity in UVB *plus* heat treated cells. In this study, we were particularly interested in the overall and persistent effects of repeated UVB *plus* heat, in order to assess potential outcomes of these exposures. To better define the pathways directly involved in UVB *plus* heat-mediated cell survival, molecular mechanisms need to be assessed at earlier stages of the cellular stress response. In addition, while the use of ex vivo skin preserved the close interaction of keratinocytes and melanocytes as an epidermal-melanin unit, which ensures protection against UVB and other stressors [53], and provided for accurate measures of clinically relevant changes in keratinocytes after UVB and/or heat exposures, this study was largely disadvantaged by small sample size. This limitation will also need to be addressed in future studies in order to concretely define the effects of UV and heat exposure on keratinocyte biology, particularly in defining heat-induced alterations in the genetic or molecular profiles of these cells.

## Conclusions

In conclusion, this study showed for the first time that multiple exposures to heat stress, in addition to UVB, prevents DNA-damaged human keratinocytes from undergoing apoptosis, as a result of inactivation of the p53 function. The results suggest that exposure to UVB and heat stress may act synergistically to allow survival of damaged cells, which could have implications for initiation of skin carcinogenesis. Knowledge of the effects of UVB *plus* heat stress on skin carcinogenesis can be utilised to decrease risk exposures particularly for people exposed to combinations of these environmental hazards in workplaces such as the mining, construction and petroleum industries.

## Abbreviations

CPD, cyclobutane pyrimidine dimers; IPA, ingenuity pathway analysis; NHEK, primary normal human epidermal keratinocytes.

## Additional files

Additional file 1: Figure S1. Immunohistochemical staining of cytokeratin (CytoK) or CPD (red), p53 or active caspase-3 (green) and DAPI (blue) in untreated or UVB and/or heat treated NHEK or ex vivo skin. Inset images are an enlarged view of CPD/p53 positive and CytoK/Casp-3 cells. Arrows indicate cells expressing CPD/p53 (orange) and CytoK/Casp-3 (white). H and E staining of untreated or UVB and/or heat treated ex vivo skin. Broken lines denote the epidermal/dermal border. Scale bar (white line) =100 μm. (JPG 713 kb) Additional file 2: Figure S2. (a) Immunohistochemical staining of SIRT1-p (green), total SIRT1 (red) and DAPI (nucleus, blue) in skin samples or primary keratinocytes that were either untreated, or exposed to heat, UVB or UVB plus heat. Broken lines denote the epidermal/dermal border. Scale bar (white line) =100 μm. Inset images are enlarged view of SIRT1/SIRT1-p positive cells, which are also indicated by red arrows. (b) Bar graphs of mean ± SD percent keratinocytes carrying phosphorylated and normal SIRT1 protein in ex vivo skin. (JPG 367 kb) Additional file 3: Figure S3. Exposure to UVB plus heat induced a significant decrease in acetylated p53 levels in NHEK and in skin models. Immunohistochemical staining of CPD (red), p53-a382 or SIRT1-p (green) and DAPI (blue) in untreated or UVB and/or heat treated NHEK. Cells co-expressing p53-a382/CPD are also indicated by green arrows. Scale bar (white line) =100 μm. (JPG 304 kb)
