# Supplementary figures and images for: Heat-mediated reduction of apoptosis in UVB-damaged keratinocytes in vitro and in human skin ex vivo
Source: BMC Dermatol. 2016 May 26;16:6. doi: 10.1186/s12895-016-0043-4 (PMC4882820; doi:10.1186/s12895-016-0043-4)

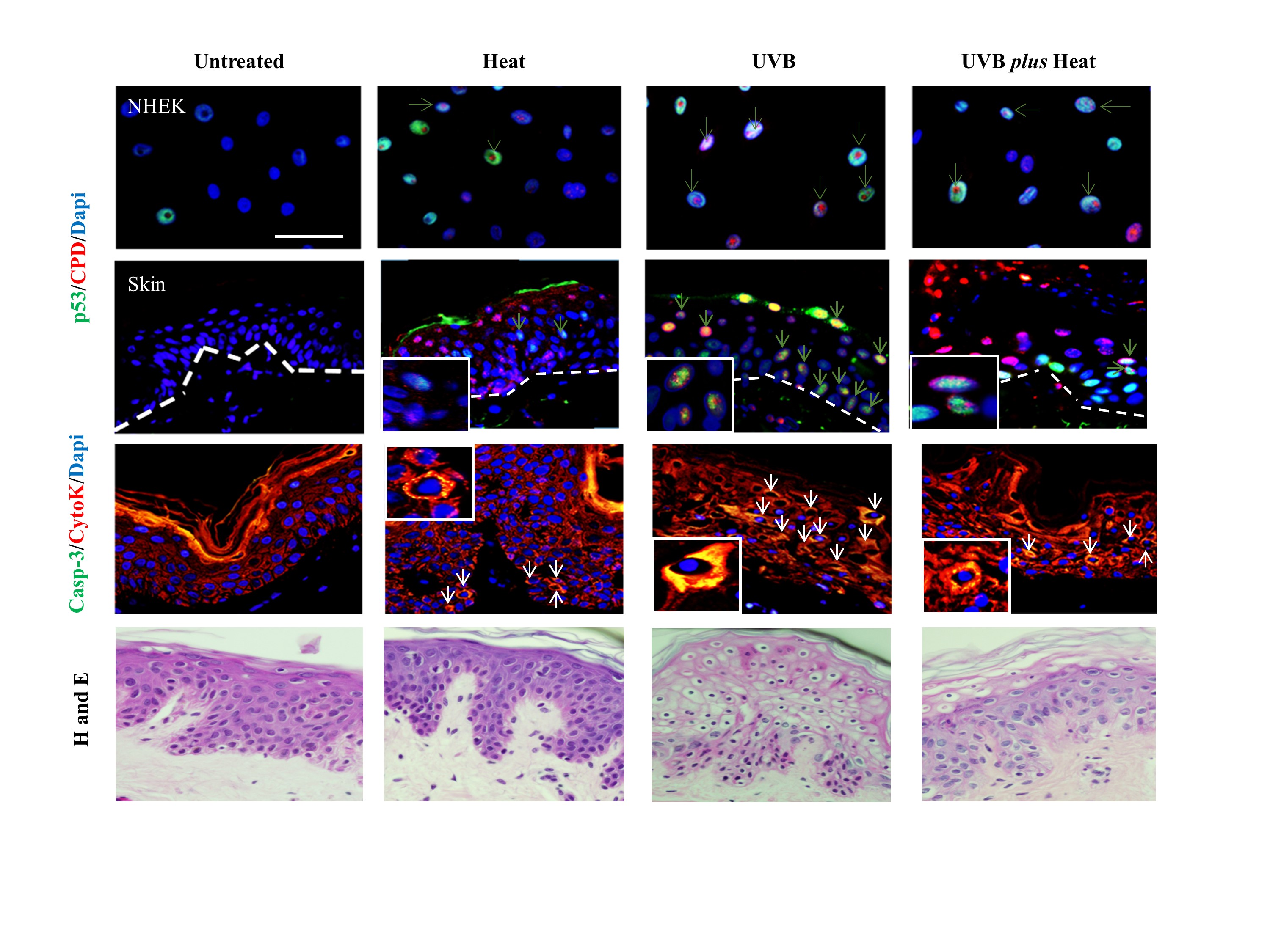

Supplement: Additional file 1: Figure S1. — Immunohistochemical staining of cytokeratin (CytoK) or CPD (red), p53 or active caspase-3 (green) and DAPI (blue) in untreated or UVB and/or heat treated NHEK or ex vivo skin. Inset images are an enlarged view of CPD/p53 positive and CytoK/Casp-3 cells. Arrows indicate cells expressing CPD/p53 (orange) and CytoK/Casp-3 (white). H and E staining of untreated or UVB and/or heat treated ex vivo skin. Broken lines denote the epidermal/dermal border. Scale bar (white line) =100 μm. (JPG 713 kb) [file 12895_2016_43_MOESM1_ESM.jpg]

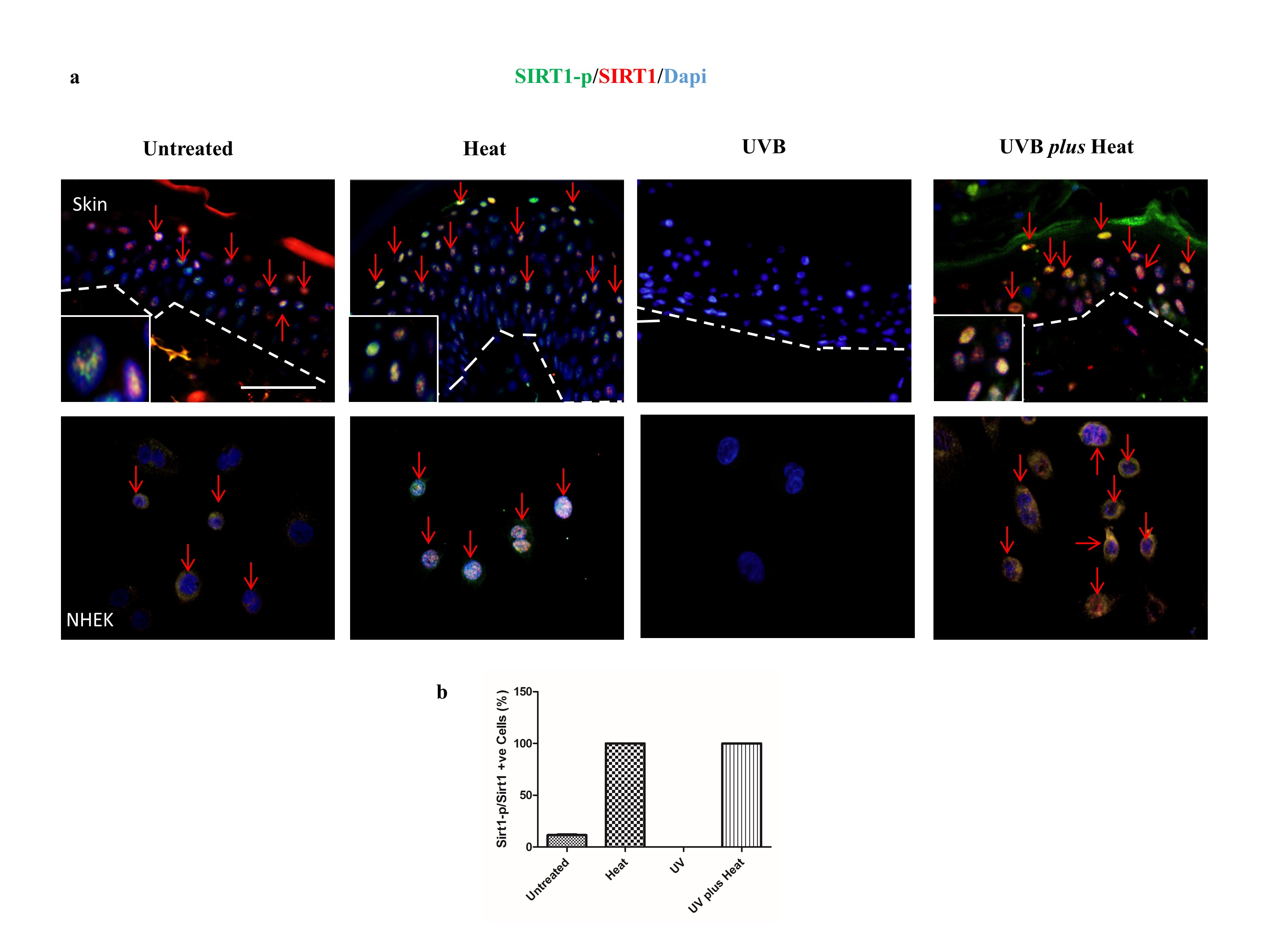

Supplement: Additional file 2: Figure S2. — (a) Immunohistochemical staining of SIRT1-p (green), total SIRT1 (red) and DAPI (nucleus, blue) in skin samples or primary keratinocytes that were either untreated, or exposed to heat, UVB or UVB plus heat. Broken lines denote the epidermal/dermal border. Scale bar (white line) =100 μm. Inset images are enlarged view of SIRT1/SIRT1-p positive cells, which are also indicated by red arrows. (b) Bar graphs of mean ± SD percent keratinocytes carrying phosphorylated and normal SIRT1 protein in ex vivo skin. (JPG 367 kb) [file 12895_2016_43_MOESM2_ESM.jpg]

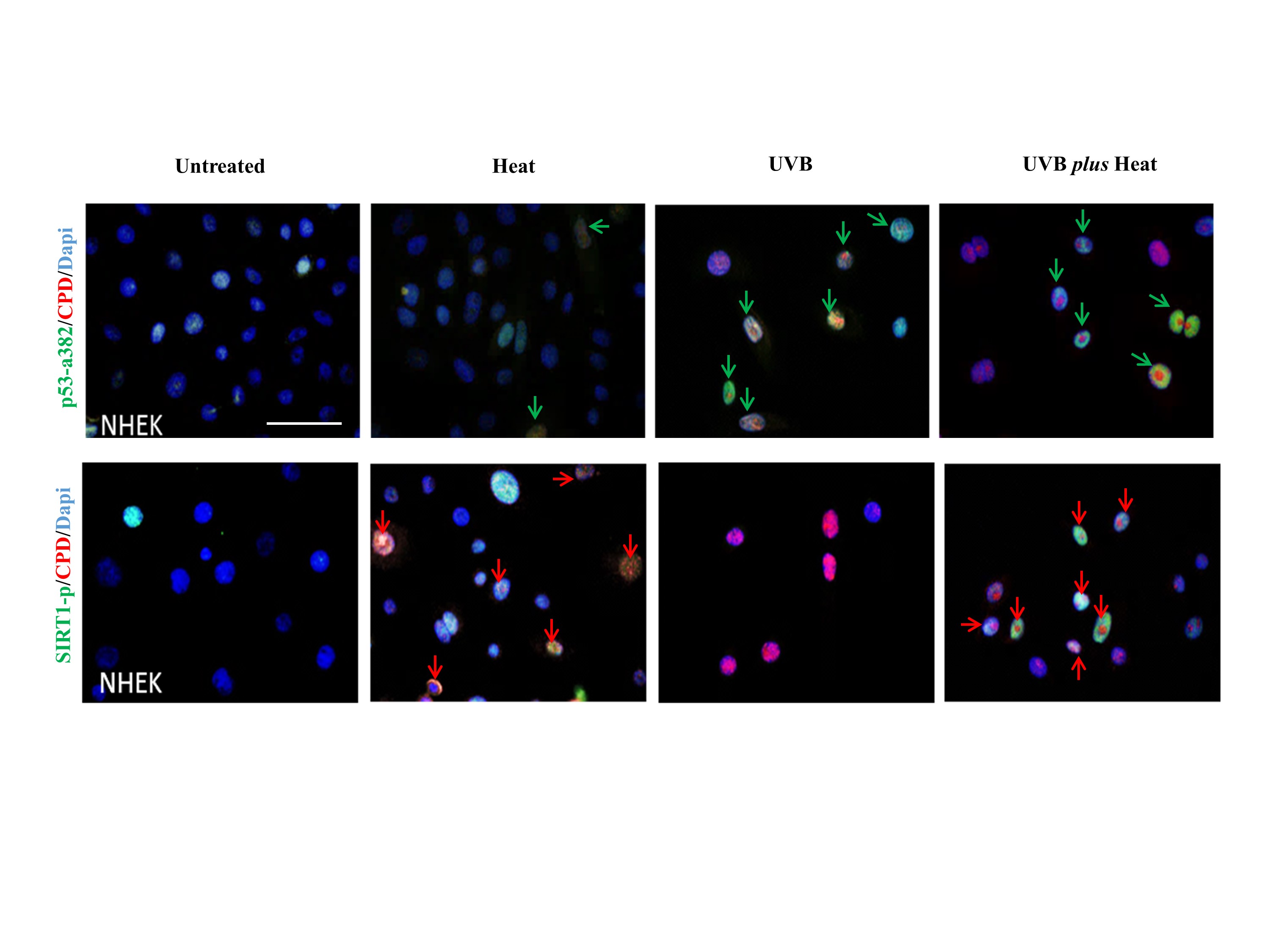

Supplement: Additional file 3: Figure S3. — Exposure to UVB plus heat induced a significant decrease in acetylated p53 levels in NHEK and in skin models. Immunohistochemical staining of CPD (red), p53-a382 or SIRT1-p (green) and DAPI (blue) in untreated or UVB and/or heat treated NHEK. Cells co-expressing p53-a382/CPD are also indicated by green arrows. Scale bar (white line) =100 μm. (JPG 304 kb) [file 12895_2016_43_MOESM3_ESM.jpg]
